# Responses to interocular disparity correlation in the human cerebral cortex

**DOI:** 10.1111/opo.12121

**Published:** 2014-02-21

**Authors:** Ifan Betina Ip, Loredana Minini, James Dow, Andrew J. Parker, Holly Bridge

**Affiliations:** 1 Department of Physiology, Anatomy & Genetics, University of Oxford, Oxford, UK; 2 Centre for Functional Magnetic Resonance Imaging of the Brain (FMRIB), University of Oxford, Oxford, UK; 3 Department of Experimental Psychology, University of Oxford, Oxford, UK

**Keywords:** binocular disparity, correspondence problem, fMRI, interocular disparity correlation, multivariate pattern analysis, stereopsis, V3

## Abstract

**Purpose:**

Perceiving binocular depth relies on the ability of our visual system to precisely match corresponding features in the left and right eyes. Yet how the human brain extracts interocular disparity correlation is poorly understood.

**Methods:**

We used functional magnetic resonance imaging (fMRI) to characterize brain regions involved in processing interocular disparity correlation. By varying the amount of interocular correlation of a disparity‐defined random‐dot‐stereogram, we concomitantly controlled the perception of binocular depth and measured the percent Blood‐Oxygenation‐Level‐Dependent (%BOLD)‐signal in multiple regions‐of‐interest in the human occipital cortex and along the intra‐parietal sulcus.

**Results:**

A linear support vector machine classification analysis applied to cortical responses showed patterns of activation that represented different disparity correlation levels within regions‐of‐interest in the visual cortex. These also revealed a positive trend between the difference in disparity correlation and classification accuracy in V1, V3 and lateral occipital cortex. Classifier performance was significantly related to behavioural performance in dorsal visual area V3. Cortical responses to random‐dot‐stereogram stimuli were greater in the right compared to the left hemisphere.

**Conclusions:**

Our results show that multiple regions in the cerebral cortex are sensitive to changes in interocular disparity correlation, and that dorsal area V3 may play an important role in the early transformation of binocular disparity to depth perception.

## Introduction

The visual system uses the images from our two eyes to reconstruct the position of objects in depth. One of the fundamental steps in recovering stereoscopic depth is to match visual features within our left and right eyes' retinal images and retrieve the small horizontal differences called binocular disparities.^1^ Before detecting these disparities, the visual system must solve a ‘correspondence problem’, a way of matching the two eyes' images to reveal which features in the left eye correspond to those in the right eye.^2^ This is a challenging process, during which false matches and unmatched features have to be discarded in favour of correct matches.

One method of investigating the correspondence problem is to employ anti‐correlated stereograms, in which features in the two eyes are negatively correlated and of opposite polarity^3^; one eye's view becomes the negative of the other. Such random‐dot‐stereograms do not usually generate the perception of depth but strikingly, neurons in V1 still respond to the disparity of anti‐correlated stimuli.^5^ The proportion of neurons responding to anti‐correlation decreases along the visual hierarchy,^7^ suggesting that responses to false matches may be eliminated in later stages of visual processing. Features are interocularly *uncorrelated* when the dots are placed in random independent locations in the left and right eyes' images so that there are no spatial matches between the two eyes’ images. Such a situation may arise in clinical conditions such as strabismus, where the eyes fail to converge on the same point in the external world. Binocularly uncorrelated images provide no disparity information and little is known of how the human brain responds to this form of binocular input.

Here we investigated the performance of observers on a two alternative forced‐choice task (2‐AFC) dynamic random‐dot‐stereograms (RDS) while parametrically varying disparity correlation levels. We also characterised the representation of disparity correlation in the human brain, examining the link between cortical activity and behavioural responses using Blood‐Oxygenation‐Level‐Dependent (BOLD) imaging of the cerebral cortex. Both univariate and multivariate approaches were employed to detect differences in neural responses to disparity correlation levels, with a particular focus on occipital and parietal regions that are known from earlier work to respond to stereoscopic depth.

Our results significantly extend and support behavioural observations on interocular disparity correlation^10^ and provide novel evidence that disparity correlation levels are represented in a graded manner in the human brain. The univariate results have been previously published in the conference proceedings of the International Conference on 3D Imaging (2012).

## Methods

### Participants

Ten healthy observers (two male, aged 20–38) with normal or corrected to normal vision and normal binocular depth perception (stereo‐acuity ≤ 120 arc sec disparity, as measured with the TNO‐Stereotest), who had provided written and informed consent (approved by Berkshire Ethics Committee 10/H0505/39), took part in the study. Of these, seven observers were naïve to the purpose of the experiment and three were authors. Each participated in two fMRI‐sessions, a 1‐h fMRI‐session to collect data for the main experiment to test the cortical responses to disparity correlation and an additional session to collect visual field maps using retinotopic procedures. One participant (Pt 3) was excluded because of a data acquisition error.

### Experimental paradigm

**Figure 1:**
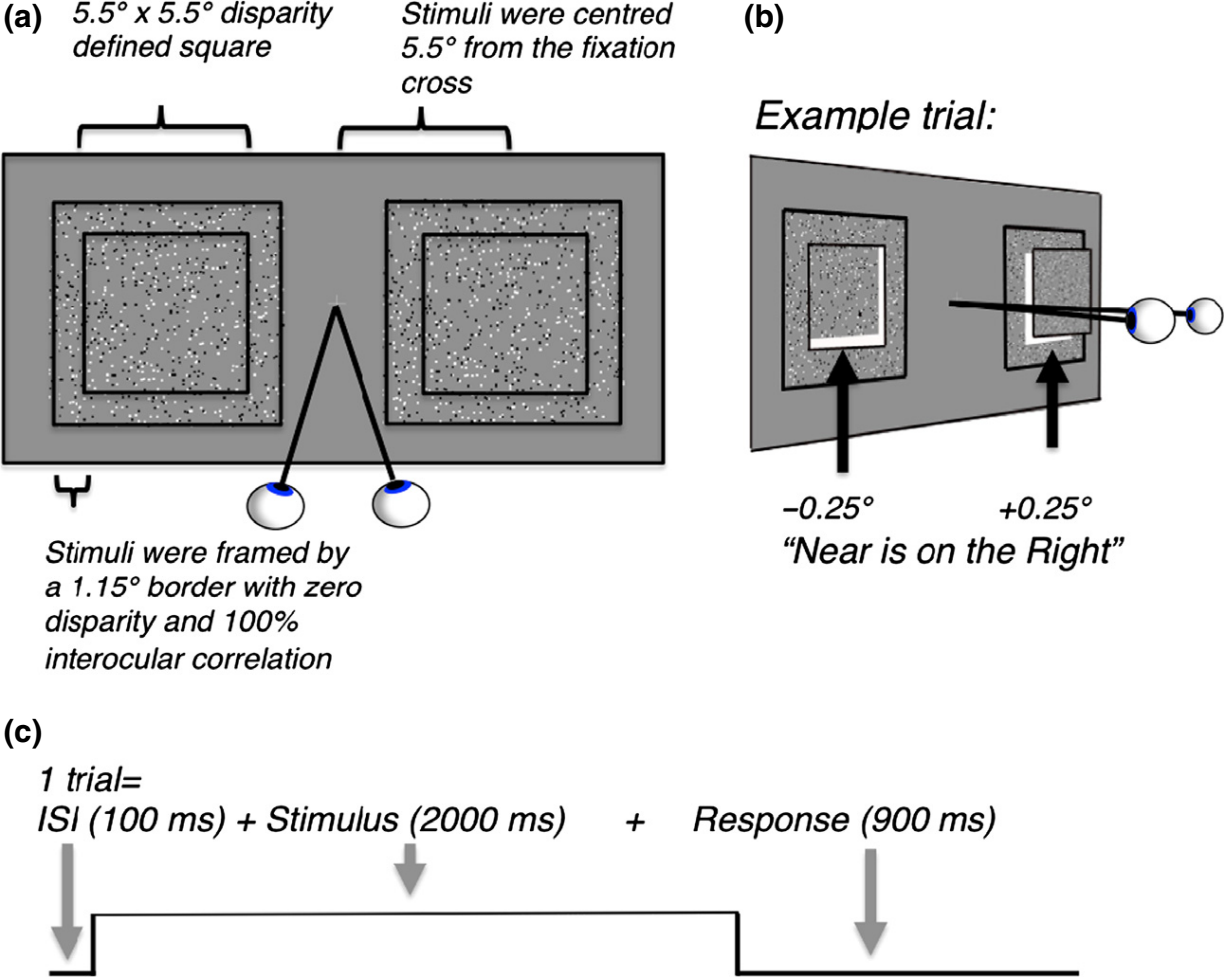
(a) schematic diagram of the stimuli. Stimuli were two square dynamic random‐dot‐stereograms (5.5 × 5.5°), plotted with ±0.25° retinal disparity and surrounded by a zero disparity frame (1.15°). Stimuli appeared as depth‐defined squares, windowed by a zero‐disparity frame. (b) opposite and equal disparities (±0.25°) were added to the two stereograms, resulting in one stimulus at a ‘near’ and one at a ‘far’ position on every trial. Subjects pressed a button corresponding to the side on which the ‘near’ patch was perceived. Illustration shows an example trial in which the left stimulus is plotted with a far disparity and the right with a near disparity. (c) diagram of the trial structure. Each trial was composed of a sequence of events: a blank grey screen with a central fixation cross (100 ms), followed by two RDS‐stimuli (2000 ms) and a response period (900 ms). Each trial lasted 3000 ms.

*Figure* 1*a* shows visual stimuli as they appeared to the observer. Experimental stimuli were two square dynamic random‐dot‐stereograms (RDS) on a grey background, each composed of a 5.5 × 5.5° square of 800 white and black dots (400 dots for each eye, 0.05° size), which randomly changed location within the square at 12.5 Hz. The RDS‐stimuli were centred ±5.5° left and right of a central fixation cross and each was surrounded by a frame (1.15° width) made up of zero‐disparity random dots. The appearance of the stimulus was that of a depth defined square windowed by a zero‐disparity frame. Binocular depth perception was experimentally tested by changing the percentage of correlated dots in the left and right eyes. At 60% disparity correlation, 60% of the dots that defined the squares were plotted with a ±0.25° disparity, whilst the remaining 40% were plotted at unrelated locations. Hence, at this correlation level, only 60% of all dots could be binocularly fused to provide stereoscopic depth information; the remaining dots were randomly plotted at uncorrelated positions and were diplopic and could not be fused. To an observer with intact stereoscopic depth perception, stimuli with high correlation appeared as fronto‐parallel disparity‐defined squares, with one square plotted at a ‘near’ depth and one at a ‘far’ depth with respect to the zero‐disparity fixation cross and the zero‐disparity frame, as illustrated in *Figure *1*b*.

Whether the left patch was presented with near or far disparity was randomly changed between trials; the right patch was always the opposite disparity from the left. Stimuli with low disparity correlation appeared as a cloud of dots with little or no depth. During the experiment, participants were instructed to keep their eyes fixed upon the central fixation cross at all times and to report whether the patch which appeared nearer to them had been presented on the left or right side of the fixation cross. During baseline blocks, subjects were presented a blank grey screen with a fixation cross and instructed to maintain passive fixation. In practice experiments before the MRI‐scan, observers were invited to practise the task on a laptop that displayed stereoscopic stimuli using anaglyph (red‐green) glasses. Participants performed the task until they reached at least 75% correct performance at the highest disparity correlation level presented in the MRI‐scanner (this was a correlation level of 60%). Random responding during the task would result in chance performance (50% correct). In the MRI‐scanner, a run consisted of 25 blocks, with each block consisting of 10 × 3 s trials of one out of five conditions (5%, 20%, 40% and 60% disparity correlation levels and one baseline condition composed of a blank grey screen with a fixation cross). Blocked conditions were pseudo‐randomised within the run (total duration 750 s). The sign of the disparity for left and right stimuli was randomly changed between trials within each block; hence cortical responses to blocked conditions provided no information specific to the sign of disparity.

### Functional MRI

Echo‐planar images (resolution of 2.5 × 2.5 mm^2^, 42 × 2.5 mm slices, repetition time (TR) = 3 s) were used to measure the blood‐oxygenation‐level‐dependent BOLD signal^12^ using a 3T Siemens Trio scanner (Oxford Centre for Magnetic Resonance, www.ocmr.ox.ac.uk/home) equipped with a 12‐channel coil. 250 volumes were collected on each run and three individual runs were collected per participant in the same experimental session, adding up to a total of 750 volumes/participant. In addition, a T1‐weighted scan (resolution of 1 × 1 mm^2^ voxels, 192 × 1 mm slices, TR = 2 s, TE = 4.7 ms) optimised for grey and white matter separation was collected.

Stimuli were projected onto a black acrylic glass back‐projection screen (ST‐Professional‐DC, Screen‐Tech, www.screen-tech.de) in the MRI‐scanner room using a CHRISTIE Mirage S + 2K projector (100 Hz, 1400 × 1050 pixel resolution). Binocular disparity was displayed by placing a ZScreen (RealID StereoGraphics, www.reald.com) in front of the projector lens that alternately circularly polarised light from each video frame (50 Hz) to clockwise or counter‐clockwise directions. Subjects wore polarised goggles and viewed stimuli via a mirror attached to the head coil. The experimental paradigm was programmed using custom‐made software and displayed using an OpenGL stereo‐system powered by an NVIDIA Quadra FX 1400 Graphics card. Behavioural responses were given using a MRI‐safe button‐box.

### Definitions of visual areas and intraparietal sulcus regions‐of‐interests

Retinotopy stimuli were presented using a VSG 2/5 graphics card (Cambridge Research Systems, www.crsltd.com) and projected with a XGA projector (Sanyo, www.us.sanyo.com) on a screen mounted on the rear of the MRI‐scanner bore. Standard retinotopic mapping procedures were followed to delineate visual areas in individual observers.^13^ A motion defined rotating‐wedge stimulus was used to map the polar angle representation of the visual field in individual subjects.^3^ The stimulus was composed of a circular field of 500 black and white static dots with a central fixation cross on a white background. A 90‐degree section of the circle formed a wedge pointing towards the fixation cross and rotated through the circular field around the fixation cross, changing rotation by 45° on every excitation pulse repetition (TR = 3 s) (3 s at each position, 8 positions, 24 s for a cycle, 10 cycles per scan). An average polar angle map was constructed by averaging across seven acquired rotating‐wedge scans. For a subset of participants, the polar angle representation was mapped using a contrast reversing (8 Hz) black and white checkerboard‐wedge presented on a grey background with a small red central fixation dot. A 45‐degree wedge with the tip pointing towards the fixation dot traversed the visual field by rotating 30° every TR (4 s) (4 s at each position, 12 positions, 48 s for a cycle, 6 cycles per scan). Four runs were averaged to construct a polar angle map. The selectivity of a voxel to a particular location in the visual field was assessed using a coherence metric that is calculated by dividing a voxel's response to a location in the visual field by responses to the summed amplitude of all other voxels. The resulting colour‐coded maps were used to define visual areas. Visual areas were delineated for each hemisphere separately and then combined across dorsal and ventral representations for early visual areas (V1‐V3). Retinotopy data were analysed with the mrVista package (http://vistalab.stanford.edu/newlm/index.php/MrVista) and manually mapped onto computationally flattened surfaces generated using the SurfRelax software.^15^

fMRI data were collected using a standard echo‐planar imaging sequence (motion‐defined wedge: resolution of 2.5 × 2.5 mm^2^, 42 × 2.5 mm slices, TR = 3 s; luminance‐defined wedge: resolution of 2 × 2 mm^2^, 42 × 2 mm slices, TR = 2 s) with coronal slices oriented perpendicular to the calcarine sulcus. A reduced field‐of view T1‐weighted image (resolution: 1 × 1 mm^2^, 40 × 2 mm slices) in the same orientation as the EPI‐scan was collected to aid registration between functional and high‐resolution anatomical images. Seven ROIs in the visual cortex (V1‐V3, V3a/b, hV4, hMT+, V7) were identified in each hemisphere of each participant using standard phase‐coded retinotopic mapping methods^14^ and anatomical landmarks. Definition of V3a^18^ may have included regions of adjacent area V3b,^19^ hence is denoted as V3a/b. hMT+ refers to a motion sensitive complex on the dorso‐lateral surface of the occipital cortex composed of area hMT and associated area MST.^13^ We used the Juelich histological mask of hMT/V5^20^ to compare individual hMT+ masks in the current study with V5/hMT locations from post‐mortem brains. hMT+ masks were transformed into standard space, binarised, and the resulting group mask compared to the histologically defined V5/hMT mask. On average, 52.3 ± 0.03% of voxels from individual subjects’ hMT+ masks overlapped with the histologically defined V5/hMT mask. This comparison confirmed that hMT+ mask locations shared common cortical surface areas with the histologically identified V5/hMT positions.

The object‐selective region LOC was defined using an object localizer^21^ in a separate set of eight subjects. The experiment was composed of five 16 s blocks of coloured and intact objects, which were interleaved with five 16 s blocks of the same objects but scrambled. A group statistical mask was created using FSL mixed‐effects analysis, with a cluster correction at *p* = 0.05. The minimum *z*‐statistic thresholds for the left and right LOC maps were then increased for left and right masks independently, until the remaining volumes (right LOC = 2108 mm^3^, left LOC = 2024 mm^3^) were comparable to the average volume of LOC masks (2027 mm^3^ ± 236 S.E.M.) reported by Sayres and Grill‐Spector.^22^ Visual inspection confirmed that LOC masks were located bilaterally, along the lateral occipital surface, posterior and inferior to hMT+ and overlapping partially with the inferior aspect of the hMT+ region‐of‐interest (ROI).^22^ In addition, spherical ROIs of 6 mm diameter located along the intraparietal sulcus were added. These were dorsal IPS medial (DIPSM), dorsal IPS anterior (DIPSA) and putative human anterior intra‐parietal region (phAIP), centered on previously reported MNI‐coordinates reported by Georgieva et al.^24^

### Data analysis

#### Psychophysical response

Psychophysical responses were quantified as ‘Proportion Correct’ and averaged across participants (*N* = 10). Group averaged data were fitted with a cumulative Gaussian and the psychometric threshold was estimated as the % disparity correlation that equated to a performance of 0.75 Proportion Correct on the fitted curve.

To relate behavioural performance as closely as possible to classifier accuracy, we calculated Δ Proportion Correct. Δ Proportion Correct quantifies differences in behavioural performance between conditions. For example, a subject who shows no difference in behavioural performance between 5% and 20% disparity correlation would provide a Δ Proportion Correct of 0, although there may be sufficient stimulus‐related change to provide an above‐chance classification based on the cortical responses (Δ Proportion Accuracy). Parametric comparison of each correlation level provided six comparisons (see Table 1 for corresponding comparisons used for calculating Δ Disparity Correlation); hence we calculated six Δ Proportion Correct values. Each Δ Proportion Correct value represents the difference in behavioural performance between the respective maximum and minimum disparity correlation level. For example, to obtain Δ Proportion Correct at 15% Δ Disparity Correlation, behavioural performance at 5% disparity correlation was subtracted from performance at 20% disparity correlation. We calculated separate Δ Proportion Correct values for the two instances of 20% Δ Disparity Correlation (20 vs 40 and 40 vs 60% disparity correlation). As the experimental data were derived from a relatively small sample, we applied the Monte Carlo method to simulate behavioural performance from each observer on the 2‐AFC task. On each iteration, a random binomial distribution with a size of 150 and a probability corresponding to the average behavioural performance of a subject at one out of 4% disparity correlation levels was simulated. Simulations were generated using the rbinom function from the statistical distribution package R. Data from 10000 iterations were combined to yield a simulated distribution with a mean and standard deviation. Classifier accuracy and Δ Proportion Correct were *z*‐normalised within participant before further analysis was applied.

**Table 1:** All parametric binary combinations of 5%, 20%, 40% and 60% disparity correlation that were analysed in a linear classification analysis

A	B	Δ Disparity Correlation
% Disparity	Correlation	[% Disparity Correlation]
5	20	15
5	40	35
5	60	55
20	40	20
20	60	40
40	60	20

#### fMRI analysis

FSL 4.0 (www.fmrib.ox.ac.uk/fsl) and associated packages were used to analyse fMRI‐data. Pre‐processing of data included automatic correction of head‐movement,^25^ removal of low‐frequency noise and slow drift,^26^ dividing each image by the mean image intensity. Local autocorrelation correction^27^ was used for statistical analysis on voxel time‐series. Statistical significance was represented as thresholded *z*‐statistic maps (*z*‐stat > 2.3) which showed activation clusters surviving a significance threshold of *p* < 0.05.^28^ FLIRT registration was used for aligning functional volumes to the high‐resolution T1‐image.^25^ BOLD‐activation was measured in visual areas from left and right hemisphere separately. Alpha levels for statistical tests were corrected for multiple comparisons using Bonferroni‐correction.

#### Multivariate pattern analysis

In multivariate pattern analysis (MVPA), a machine‐learning algorithm is trained to detect any reliable difference between two conditions based on their assigned condition labels in a set of training data. To test whether any differences were present, the algorithm must predict the condition to which a novel set of data belongs using the learned differences from the training data. If enough information were available, the classifier would be able to predict the condition of a novel set of data with an accuracy above chance. The higher the classification accuracy, the greater is the reliability of the information. On the other hand, with no reliable difference, classification accuracy would be at chance.

MVPA was performed using standard methods.^30^ For each participant, functional runs were registered to the mean first functional volume using automatic registration (FLIRT) with 7‐degrees‐of‐freedom. A fixed‐effects analysis identified a thresholded *z*‐statistic map (*z*‐stat > 2.3) showing clusters surviving a significance threshold of *p* < 0.05 to all stimulus conditions compared to a baseline composed of a blank screen with a fixation cross. The 100 most strongly modulated voxels within each ROI (V1, V2, V3, V3a/b, hV4, hMT+, V7, LOC), separately mapped in left and right hemispheres, were selected using custom written scripts. Co‐registered functional volumes were treated as data collected in a single scan.

A binary classification using a linear classifier (LIBSVM www.csie.ntu.edu.tw/~cjlin/libsvm/, Matlab Implementation) with 7‐fold hold‐one‐out cross‐validation using individual trials was performed on each participants’ data. Accuracy was reported as the mean proportion accuracy across all participants. Classification accuracy was assessed using 95% binomial confidence intervals and corrected for multiple comparisons: classification accuracy was taken as significant at an alpha level of *p* < 0.05, if lower error bar was above the chance line (0.5 proportion accuracy). As a control for potential confounds caused by temporal order within each run, we performed a control classification in which we drew equal samples of each condition from the first and second halves of the experimental runs. For example, in the linear comparison between 5% and 60% disparity correlation, 50% of each class would be drawn from the first half and the rest from the second half of the experimental runs. We then compared results from the control with the original analysis using an N‐way anova with interaction. Consistent with results obtained for the original analysis, we found highly significant effects of ROI type (*F*_7,864_ = 6.29, *p* < 0.001) and Δ Disparity Correlation (*F*_5,864_ = 20.12, *p* < 0.001), and no significant effects of temporal order or interaction on classification results.

## Results

### Behavioural performance in the MRI‐scanner

**Figure 2:**
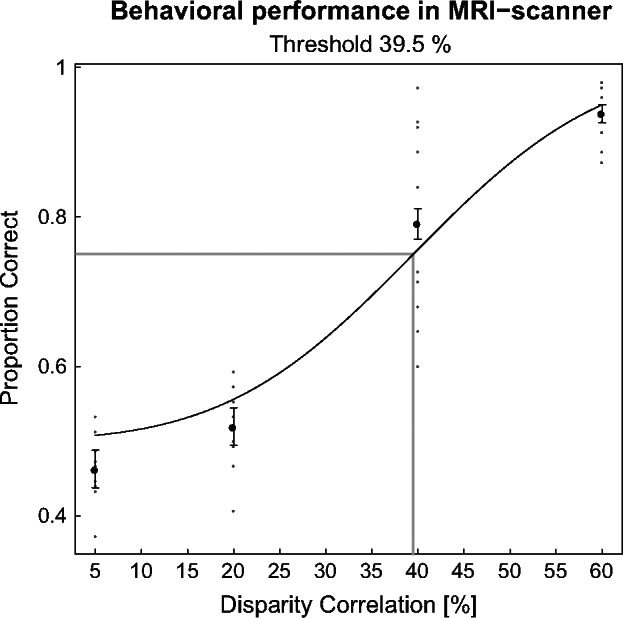
Group performance on judging the depth of two dynamic random‐dot‐stereograms at different disparity correlation levels in the MRI‐scanner. The *y*‐axis plots ‘Proportion Correct’ judgments. The *x*‐axis plots disparity correlation values. Black dots are averaged data points across subjects. Grey dots are single subjects. Black line is the cumulative Gaussian fitted to the data. Grey line indicates the threshold at 0.75 Proportion Correct. Errors bars are 95% binomial confidence intervals across observers (*N* = 10).

Observers performed the disparity task in the MRI‐scanner (see *Figure *2). The proportion of correctly identified trials increased with disparity correlation. Subjects were performing close to chance when 5% or 20% of the dots were correlated. Threshold performance (0.75 Proportion Correct) was reached when 39.5% of the dots were correlated. Our results confirm that manipulating disparity correlation was effective at controlling stereoscopic depth perception in the MRI‐scanner.

### The average BOLD‐response above baseline is not modulated by disparity correlation

**Figure 3:**
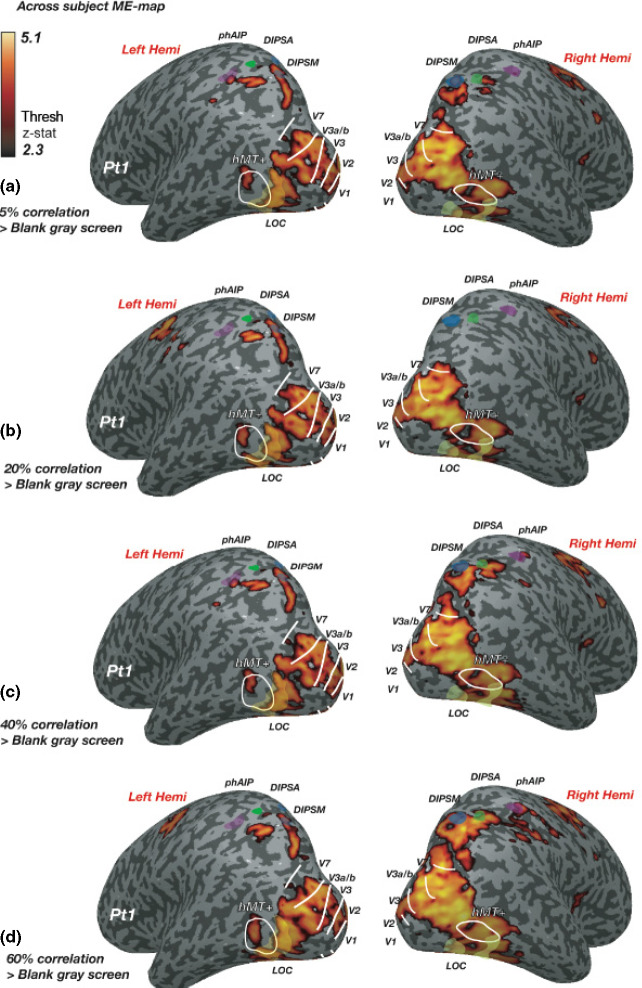
Increased BOLD response across the visual cortex in response to two disparity‐defined RDS during performance of a binocular detection task. Images show computationally inflated and flattened right and left hemispheres from a representative participant (Pt 1). (a) Statistical maps show group responses to performing a task on two RDS‐patches with 5%, at (b) 20%, (c) 40% and (d): 60% disparity correlation compared to a baseline composed of a blank grey screen with a fixation cross. White lines indicate the borders of visual areas; transparent yellow regions indicate the position of the ROI in the lateral occipital cortex (LOC). Transparent blue region = DIPSM; transparent green = DIPSA and transparent purple = phAIP. The dark grey regions on the map are sulci and light grey regions gyri.

**Figure 4:**
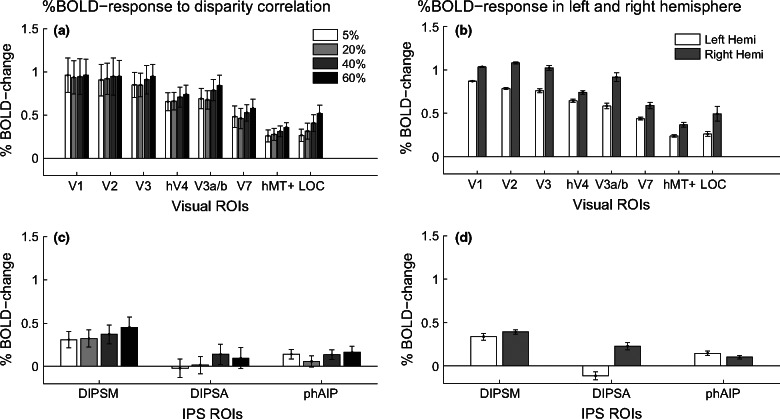
(a) and (c) show the percent BOLD change to each of the 4 disparity correlation levels relative to fixation of a grey screen in the occipital and parietal ROIs respectively. The shading of the bars indicates the disparity correlation level, with darker shades representing higher disparity correlation. (b) and (d) indicate the percent BOLD change averaged across all correlation levels, but separated by hemisphere. The white bars indicate left hemisphere ROIs and grey bars right hemisphere ROIs. Errors are ±S.E.M. across ten observers for (a) and (c) and across 4 disparity correlation levels for (b) and (d).

*Figure* 3 shows the results of a mixed‐effects group statistical map (*z*‐threshold >2.3, cluster corrected at *p* = 0.05), generated by presenting two disparity‐defined RDS‐patches at different disparity correlation levels, compared to a grey screen with a fixation cross. Stimuli caused strong modulation of cortical responses across left and right occipito‐parietal regions, overlapping ROIs in the visual cortex (V1‐V3, hV4, V3a/b, V7, hMT+), the lateral occipital cortex (LOC) and the dorsal intra‐parietal area medial (DIPSM). Comparing activation from *Figure *3*a* towards D, responses along the right occipito‐parietal surface increased with disparity correlation. In contrast, responses along the left occipito‐parietal surface remained relatively similar. To quantify these differences, percent BOLD signal was extracted from all ROIs. The results are shown in *Figure *4*a,c*, which show the data for all correlation levels from occipital and parietal areas respectively. *Figure *4*b,d* show responses averaged across all correlation levels separately for left and right cortical hemispheres. An N‐way anova showed a significant main effect of cortical area (*F*_7,597_ = 31.95, *p* < 0.001) and an effect of laterality (*F*_1,597_ = 23.6, *p* < 0.001), but no effect of correlation level. For ROIs along the IPS, an N‐way anova found main effects of laterality (*F*_1,222_ = 18.13, *p* < 0.001) and cortical area (*F*_2,222_ = 7.06, *p* < 0.01), as well as a significant interaction between laterality and area (*F* = 7.06, *p* = 0.001). This last result implies that the modulation of BOLD activity differed depending on the hemisphere in which the ROI was located. Taken together these results suggest that there is a qualitative change in activity to disparity correlation, which was however not reflected in the average ROI response.

### Spatially distributed signals are informative about disparity correlation

Conventional univariate fMRI analysis quantifies whether there is a mean change in signal by averaging responses across voxels within a spatially defined cortical region‐of‐interest. In recent years, machine‐learning algorithms such as linear support vector machine classifiers have been applied to fMRI data to measure the presence of any weak but reliable, spatially distributed information within a region‐of‐interest.^30^ Hence, MVPA‐classification is sensitive to signal changes between voxels that are lost in the spatial averaging procedure performed during conventional univariate fMRI analysis. In terms of the current study, MVPA classification tests the hypothesis whether cortical regions‐of‐interest contain spatially distributed signals that change reliably with different disparity correlation levels.

A MVPA classification using linear support vector machine classifiers was applied to test in more detail whether information about disparity correlation was present in the responses between voxels. For each ROI, this analysis used the 100 voxels with the largest response to a disparity‐defined stimulus compared to fixation. The mean signal change across voxels was removed in the pre‐processing to ensure that the linear classifier was driven by spatially distributed signals between voxels rather than residual differences in the mean signal.^31^ Insufficient numbers of voxels were available in higher areas (DIPSM, DIPSA, phAIP); hence the analysis was performed only for ROIs in the visual cortex and LOC.

**Figure 5:**
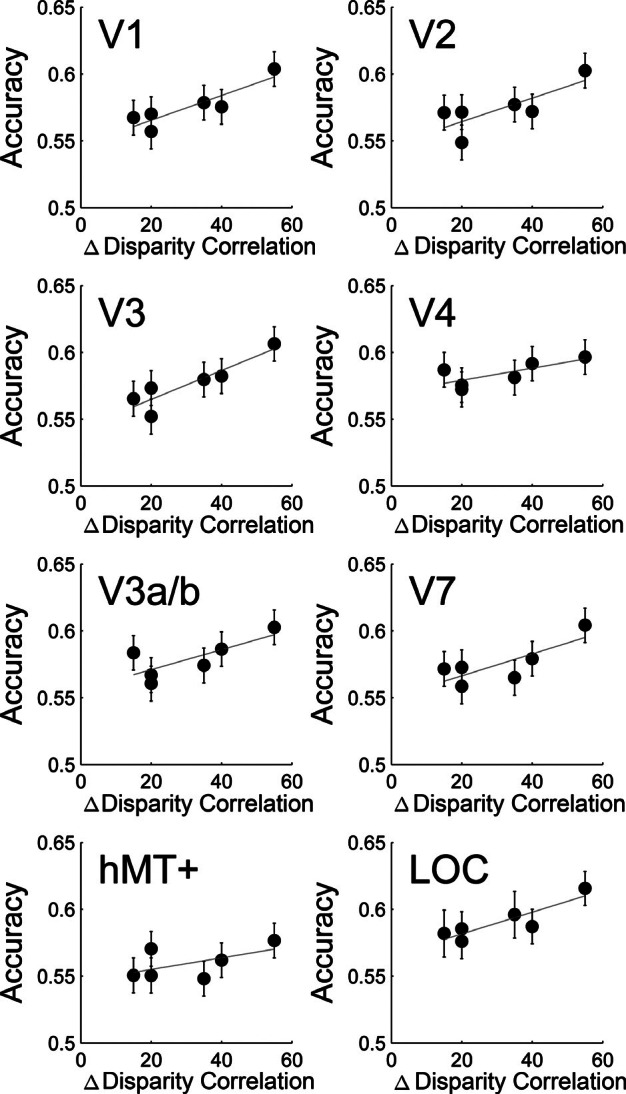
Linear regression of group classification accuracy over Δ Disparity Correlation. Error bars are binomial confidence intervals with Bonferroni‐correction. All classification results are significantly above chance, as lower error bars do not cross the chance level. Each point represents one of the six pairwise comparisons between correlation levels. Linear correlation statistics are in Table 2.

Table 1 lists all six binary combinations of disparity correlation comparisons that were used for the classification analysis. The difference between binary comparisons was quantified as Δ Disparity Correlation. *Figure *5 shows group classification results plotted over Δ Disparity Correlation. We found above chance classification in all parametric comparisons across ROIs. The classifier was sensitive to differences between 5% and 20% correlation (15% Δ Disparity Correlation), correlation levels at which most subjects still performed at chance (see *Figure *2). Across visual areas, the accuracy of the classifier rose with Δ Disparity Correlation. An N‐way anova revealed statistically significant main effects of visual ROI [*F*_7,864_ = 5.27, *p* < 0.001] and Δ Disparity Correlation [*F*_5,864_ = 17.82, *p* < 0.001], and no effects of laterality of the ROI or interaction.

Next, we applied a linear correlation analysis to further investigate the relationship between Δ Disparity Correlation and classification results (for linear correlation statistics, see Table 2). The *p*‐values in the table are not Bonferroni‐corrected. The slope measured how classifier accuracy related to Δ Disparity Correlation. A shallow slope of the classifier performance indicates that the effect of disparity correlation on classifier performance is weak (i.e. a small Δ Disparity Correlation would provide similar signal as a large Δ Disparity Correlation). A steeper slope would point towards a stronger effect of Δ Disparity Correlation on classifier accuracy. A positive relationship was found in areas V1, V3 and LOC.

**Table 2:** Linear correlation statistics of group classification accuracy and Δ Disparity Correlation. *p < 0.05

ROI	R	*F*‐stats	*p*‐value	Slope	Intercept
VI	0.806	16.595	0.015*	0.000927	0.547
V2	0.614	6.351	0.065	0.000881	0.547
V3	0.818	17.949	0.013*	0.001082	0.543
hV4	0.547	4.838	0.093	0.000455	0.570
V3a/b	0.573	5.362	0.082	0.000748	0.556
V7	0.625	6.667	0.061	0.000820	0.550
hMT+	0.312	1.818	0.249	0.000435	0.546
LOC	0.780	14.204	0.020*	0.000813	0.565

### Classification accuracy in V3 relates to behavioural performance

Our next step was to relate perceptual performance in the depth task to cortical responses. In our first attempt, we related depth perception to cortical responses by measuring how well single subject thresholds could predict classifier accuracy in different cortical regions (data not shown). We found that high thresholds were related to high classifier accuracy in dorsal V3a/b (*p* = 0.04) and hMT+ (*p* = 0.027). In these ROIs, cortical responses of subjects that needed more % disparity correlation for depth perception provided more reliable information for the classifier. This result seemed paradoxical, as one could assume that subjects with high thresholds performed worse at greater percentage disparity correlation levels because cortical responses to binocular disparity were less reliable. However, the relationship between perceptual performance and cortical responses is more complex. One possible explanation is that subjects with high thresholds required more effort to perform the task, thereby increasing the amount of available information for the classifier by boosting sensory information with visual attention. In support, Jehee et al.^36^ have shown that modulation by visual attention increases classifier accuracy in the human visual cortex. Chandrasekaran et al.,^37^ found that the BOLD signal in fronto‐parietal ROIs increased with poorer performance on a disparity defined shape detection task, which may have been due to visual attention.

Therefore, a possible limitation of the above analysis is that behavioural thresholds are only indirectly related to classifier accuracy: behavioural thresholds measured the ability to perceive depth, while classifier accuracy measured if two disparity correlation levels could be distinguished on the basis of reliable differences between cortical responses, regardless of how those differences are generated. We therefore sought a closer behavioural metric with which we could compare performance on the depth task with differences between cortical responses. Hence, our next analysis used the binary comparisons applied already in the MVPA classification to quantify differences between behavioural performance (Δ Proportion Correct) of two disparity correlation levels. We then related Δ Proportion Correct to differences between cortical responses (classifier accuracy).

**Figure 6:**
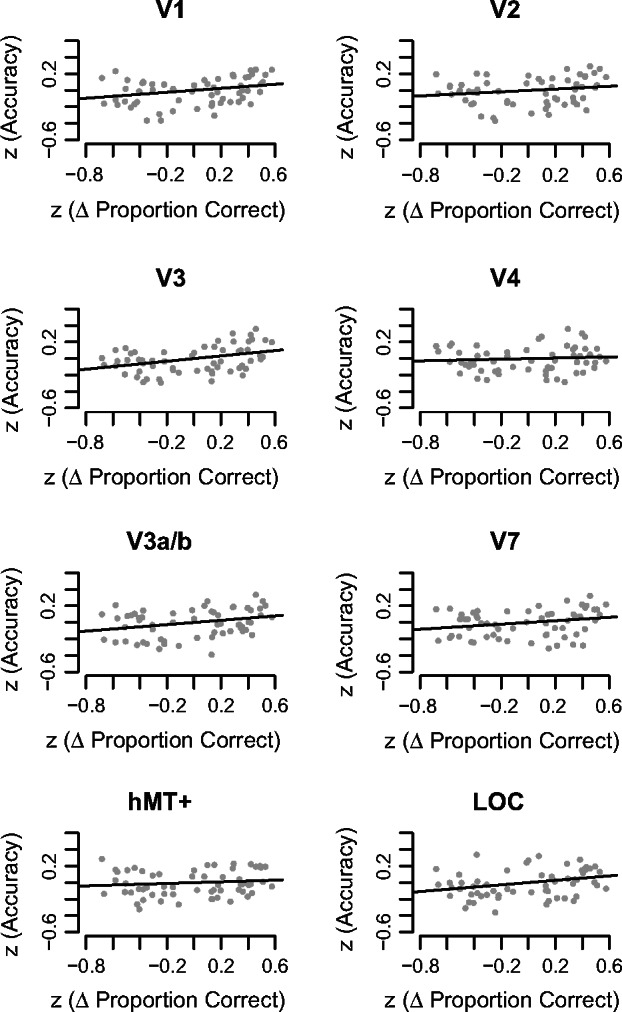
Relationship between classifier accuracy and Δ Proportion Correct measured behaviourally. The *x*‐axis represents *z*‐normalised Δ Proportion Correct, the *y*‐axis plots *z*‐normalised classification accuracy. Each grey point represents the *z*‐normalised Δ Proportion Correct from 1 out of 6 possible binary combinations of disparity correlations from 1 out of 10 subjects. Each plot shows 60 data points, composed of data from all parametric comparisons and single subjects (6 comparisons × 10 subjects).

*Figure* 6 shows the relationship between classifier accuracy and Δ Proportion Correct. To quantify this relationship, we applied a linear correlation to measure how well Δ Proportion Correct can predict classifier accuracy. We found a positive relationship in areas V1, V3, V3a/b and LOC, although only V3 survived Bonferroni‐correction at *p* < 0.05. Table 3 reports correlation statistics for these visual areas and the LOC.

**Table 3:** Linear correlation statistics relating *z*(Accuracy) to the *z*(Δ Proportion Correct). *p < 0.05, **p < 0.01

ROI	R	*F*‐statistic	*p*‐value
VI	0.082	5.149	0.027*
V2	0.037	2.228	0.141
V3	0.146	9.946	0.003**
hV4	0.007	0.420	0.519
V3a/b	0.084	5.288	0.025*
V7	0.056	3.456	0.068
hMT+	0.015	0.854	0.359
LOC	0.092	5.876	0.018*

## Discussion

We studied human visual cortical responses to different levels of interocular disparity correlation using a 2‐AFC depth‐discrimination task performed in the MRI‐scanner. We show that depth perception is linked to disparity correlation levels when performing behaviourally in the MRI‐scanner. Multivariate pattern analysis applied to BOLD signals was able to find patterns of activation that represent different levels of disparity correlation within ROIs in the visual cortex. There was a positive trend between classification accuracy and disparity correlation in V1, V3 and LOC. Furthermore, cortical responses to disparity correlation in these areas and V3a/b related to performance in the depth task. These findings suggest that information about disparity correlation is represented across multiple processing levels in the visual cortex and further implicate dorsal region V3 in processes leading to stereoscopic depth perception.

### Information about interocular disparity correlation is represented in multiple areas in the occipital cortex

Neurophysiological studies have shown that neurons sensitive to binocular disparity are organised on a fine spatial scale in the primate visual cortex. For example, strongly disparity‐selective regions in macaque area MT are interspersed with weakly tuned regions in patches of about 300–700 μm wide.^38^ Differential activity on such a scale would be lost in a univariate fMRI analysis that averages activity across an entire cortical area. To overcome this methodological limitation, multivariate classification approaches have been applied to fMRI analysis. Using these techniques, human cortical activity has been shown to contain information about direction selectivity^30^ and disparity selectivity^31^ amongst others. Our multivariate classification analysis found sensitivity to different disparity correlation levels in the primary visual area V1 as well as dorsal and ventral visual areas. The analysis also revealed significant classification accuracy when comparing the two smallest disparity correlation levels, conditions in which participants performed close to chance. Classification of these conditions is likely driven by differences in the patterns caused by stimuli based rather than perceptual differences. This result is consistent with the ability of MVPA to reveal perceptually invisible^33^ or unattended visual features.^31^ Our current results further extend the characterization of V1, V3 and LOC by showing that classification accuracy of these areas relates to differences in disparity correlation.

Recent human neuro‐imaging studies have consistently highlighted the role of dorsal retinotopic visual areas V3a/b and V7^31^ in disparity processing. We found no clear dichotomy between the classification accuracies of dorsal and ventral stream areas when comparing accuracies based on cortical responses to planar RDS‐stimuli at different correlation levels. We also applied a linear correlation analysis to test the relationship between classifier performance and disparity correlation levels and found a positive trend between disparity correlation and classifier accuracy in primary visual area V1, area V3 and ventral region LOC. Previous human neuroimaging studies have revealed univariate BOLD‐signals to detection of disparity defined shapes^37^ and multivariate signals to near/far position in depth^31^ in ventral LOC. Recent neuropsychological evidence on a patient with bilateral damage to the LOC region showed a specific deficit in performing tasks that require fine judgments on the relative position of a surface rendered in depth.^41^ The data presented here strengthen the link between ventral area LOC and disparity processing by demonstrating a graded response to disparity correlation in LOC of subjects with normal binocular vision.

### A significant positive relationship between behavioural performance and classifier accuracy in dorsal visual area V3

Our previous analysis already established that across subjects, disparity correlation was positively related to classification accuracy. As the next step, we reduced the contribution of stimulus‐related changes on the analysis by using the difference in behavioural performance as the predictor. To compare behavioural performance to accuracy, we computed Δ Proportion Correct, a metric that quantified the difference in performance level at two disparity levels. We found a significant positive relationship between V3 and Δ Proportion Correct (*p* < 0.05, Bonferroni corrected), and to a lesser extent in V1, V3a/b and LOC. This means that classifier accuracy increased as a function of Δ Proportion Correct: two correlation levels that led to the same level of performance related to a low accuracy, whereas two correlation levels that led to very different levels of performance equated to a higher accuracy.

A strong neurophysiological basis for disparity processing in primate V3 exists. Neurons in V3 respond to correlated, anti‐correlated and uncorrelated binocular disparity.^42^ Up to half of the neurons in V3 are tuned to binocular disparity,^43^ and disparity tuned neurons are organised in clusters according to disparity preference.^45^ Cortical responses in macaque V3 respond strongly to disparity defined stimuli, although this pattern was not evident in the human brain.^46^

The role of human V3 in disparity processing is less well characterised. Chandrasekaran et al.,^37^ found a positive correlation between V3 responses and detection of a disparity‐defined shape while Georgieva et al.,^24^ showed that cortical responses in V3 could discriminate disparity‐defined fronto‐parallel and curved surfaces. Interestingly, both studies used 100% interocularly correlated stimuli that were defined by a binocular disparity shape gradient, supporting a role for V3 in later stages of disparity processing. Our study is the first to suggest that human V3 may play a role in the early stages of binocular disparity processing.

### Greater activation in the right hemisphere to RDS‐stimuli

We found an increase in cortical responses to disparity correlation in the right hemisphere (*Figure *3). Quantified %BOLD‐change in the left to right hemisphere revealed a significantly greater response to RDS‐stimuli in the right hemisphere (*Figure *4*b,d*). This bias may have been due to the deployment of attention. Performing the behavioural task at any disparity correlation level would have required visual attention. Voluntary control of spatial attention activates a right hemisphere lateralised network of fronto‐parietal areas,^24^ which would modulate similar regions in the posterior parietal cortex as those mapped by the ROIs along the IPS.^49^ RDS‐stimuli at higher correlation levels may also have been perceptually more salient and attracted more visual attention. This increase in perceptual salience could also account for the greater activation in the right hemisphere to the RDS‐stimuli.

However, our results are also in line with previous observations that binocular disparity processing may occur in a right lateralised network. Early brain damage studies pointed towards a reduced ability to perceive disparity defined shapes in patients with diffuse damage to the right cerebral hemisphere^50^ and impairment in stereopsis of patients^51^ and monkeys^52^ with right temporal lobe removed. Vaina^53^ showed that right occipito‐parietal lesion patients failed completely on a stereoscopic form detection and structure‐from‐motion task, while right occipito‐temporal lesion patients retained some residual depth perception and were able to detect structure from motion. In contrast, patients with left hemisphere lesions were much less impaired in depth perception. Subsequently, fMRI studies in healthy human participants have shown that 2D structure‐from‐motion^54^ and disparity‐defined stimuli^56^ activate a predominantly right lateralised network of occipital and intra‐parietal regions.

Future studies will need to compare left and right hemisphere responses to a stereoscopic task with a difficulty matched non‐stereoscopic task to dissociate general performance advantages and attention related activity from responses specific to stereoscopic depth processing.

### Influence of uncorrelated dots and vergence eye‐movements

While our results show a significant classification result in all areas investigated, we are unable to determine definitively which sources of neural information may be driving the classifier. For example, responses in V3 may have reflected differential activation profiles due to disparity‐selective columns,^45^ whereas responses in LOC may have reflected the emergence of a disparity‐defined shape selectivity, consistent with the role of LOC in shape analysis.^37^ One important possibility we are able to exclude is that the classifier was driven primarily by the percentage of uncorrelated dots. As disparity correlation levels decrease, left and right eyes are presented with an increasing percentage of dots visible to only one eye. Conceivably, the classifier may have picked up the signal relating to the number of the uncorrelated dots, rather than the signal from the interocularly correlated dots. In this case, the most reliable signal would be at 5% correlation, as 95% of dots would be uncorrelated. Hence, classification accuracy would increase as correlation levels decrease. However, our control analysis (data not shown) demonstrated that this was not the case.

We think that it is highly unlikely that vergence or saccadic eye‐movements confounded our paradigm. Participants were instructed to keep central fixation at a zero‐disparity fixation cross at all times and the amount of binocular disparity added to the left and right stimuli was always of equal magnitude and opposite sign, balancing depth cues across the visual field. As a consequence, there is no net drive to the vergence system as the RDS‐stimuli are presented.

## Conclusion/summary

In summary, our findings measure binocular disparity processing in the human brain by characterising cortical responses to different levels of interocular disparity correlation. Our results provide evidence that several areas identifiable in the visual cortex and the LOC region represent differences in disparity correlation. Of the various cortical areas examined, V3 shows the clearest correlation between the cortical response and stereoscopic depth perception based on the data from single subjects. Future studies should investigate whether the relationship between individual subjects' cortical responses and perception can help diagnose whether individuals with abnormal binocular vision might benefit from visual therapy.
